# VGLUT2 may improve cognitive function in depressed rats by protecting prefrontal cortex neurons

**DOI:** 10.3389/fnbeh.2024.1453161

**Published:** 2024-09-05

**Authors:** Longfei Liu, Yongxue Hu, Qing Shan, Peifan Li, Tianpei Ma, Yiming Wang

**Affiliations:** ^1^College of Clinical Medicine, Guizhou Medical University, Guiyang, China; ^2^Department of Psychiatry, Affiliated Hospital of Guizhou Medical University, Guiyang, China

**Keywords:** depressive behaviors, cognitive decline, VGLUT2, CUMS, prefrontal cortex

## Abstract

**Objective:**

Depression may be accompanied by cognitive impairment, but its pathogenesis remains unclear. This study aims to investigate the protective effects of fluoxetine on behavioral performance and prefrontal cortex neuronal damage in rats with depression-associated cognitive impairment, based on the observation of VGLUT2 protein expression.

**Methods:**

Forty-five SPF-grade male SD rats were randomly divided into three groups (*n* = 15): normal control group (CON), depression group (DD), and fluoxetine group (DD + F). The CON group was reared normally, while the DD and DD + F groups underwent chronic unpredictable mild stress (CUMS) combined with social isolation to induce a depression-related cognitive dysfunction model. After modeling, the DD + F group was treated with fluoxetine (10 mg/kg, ig) for 14 days. Behavioral tests were performed to assess changes in mood, cognition, learning, and social abilities. Histopathological observations were made to examine pathological changes, neuronal apoptosis, ultrastructure, and dendritic spine density in the prefrontal cortex. The concentration, relative expression level, and mRNA expression of VGLUT2 protein were also measured. Finally, a correlation analysis was performed between the relative expression level and mRNA expression of VGLUT2 protein and the pathological changes in neurons.

**Results:**

Compared to the CON group, the DD group exhibited decreased body weight, anhedonia, increased behavioral despair, reduced locomotor activity and spontaneous exploratory behavior, impaired spatial learning and memory, and decreased social interaction and social cognitive ability. Pathological damage was observed in the prefrontal cortex, with neuronal apoptosis, ultrastructural damage, and reduced neuroplasticity. The concentration, relative expression, and mRNA expression levels of VGLUT2 protein were decreased. Following fluoxetine intervention, the above behavioral phenotypes improved; pathological damage showed varying degrees of recovery; and the concentration, relative expression, and mRNA expression levels of VGLUT2 protein increased. Finally, there was a significant correlation between VGLUT2 protein expression and pathological changes in the prefrontal cortex.

**Conclusion:**

After 28 days of CUMS combined with isolation rearing, rats exhibited impairments in mood, cognition, learning, and social abilities, with neuronal damage and decreased VGLUT2 protein levels in the prefrontal cortex. Following fluoxetine intervention, VGLUT2 protein expression increased, neuronal repair in the prefrontal cortex occurred, depressive-like behavior improved, and cognitive learning and social abilities were restored.

## Introduction

Depression is a common psychological disorder characterized by a triad of emotional, physical, and cognitive symptoms (Chinese Society of Psychiatry Chinese Academy of Depressive Disorders et al., 2020; Malhi and Mann, 2018). Among these, cognitive dysfunction is prevalent across all stages of depression (Conradi et al., 2011; Wang et al., 2019; Zuckerman et al., 2018), and its recovery is a crucial factor in achieving true clinical remission for patients with depression (Li, 2018). Cognitive dysfunction tends to worsen with increasing relapses of depression (Semkovska et al., 2019). Currently, the clinical emphasis on cognitive symptoms in depression remains insufficient, and there is a lack of specific drugs to improve cognitive symptoms in patients with depression (Keefe, 2016). Some studies have found that SSRIs can improve cognitive symptoms (Atique-Ur-Rehman and Neill, 2019; Baune and Renger, 2014; Rosenblat et al., 2015), but the mechanism remains unclear and requires further research. Research has shown a close relationship between cognitive function and excitatory neurotransmission in the prefrontal cortex (Adhikari et al., 2015). Vesicular glutamate transporter 2 (VGLUT2) is a marker protein for excitatory glutamatergic neurons, and depressive behavior is closely related to the expression of VGLUT2. Modulation of VGLUT2 can result in depressive behavior (Szőnyi et al., 2019). However, there is currently no research on the impact of VGLUT2 on cognitive dysfunction in depression.

This study utilized a combination of chronic unpredictable mild stress (CUMS) and social isolation to induce a depression model in rats, resulting in impairments in emotion, cognition, learning, and social abilities. The SSRI drug fluoxetine was selected for intervention, and the neurobehavioral, histopathological changes, and alterations in VGLUT2 in the prefrontal cortex were observed. The correlation between pathological changes in the prefrontal cortex and VGLUT2 protein was analyzed. The aim was to explore the regulatory effect of SSRI drugs on VGLUT2 in the prefrontal cortex and its impact on the behavioral and histopathological changes in rats with depression-induced cognitive dysfunction, providing more insights for the clinical treatment of cognitive dysfunction caused by depression.

## Materials and methods

### Reagents

Fluoxetine hydrochloride, specifications: 20 mg*28 tablets (Eli Lilly Suzhou Pharmaceutical Company); The neurobehavioral apparatus was purchased from Shanghai Xinruan Information Technology Co., Ltd.; VGLUT2 antibody (catalog number: ab216463); Large-scale instruments used in the experiment were provided by the Clinical Research Center of the Affiliated Hospital of Guizhou Medical University.

### Animals model

Forty-five adult male healthy Specific Pathogen Free (SPF) Sprague–Dawley (SD) rats, with a body weight ranging from 220 to 270 grams and an age of 6 weeks, were purchased from the Experimental Animal Center of Zunyi Medical University, with the experimental animal use permit number: SCXK (Qian) 2021–0002. The breeding conditions were set at a temperature of (22 ± 2)°C, relative humidity of (50 ± 10)%, and a light–dark cycle of 12 L/12D (7.00–19:00) to maintain the animals’ normal circadian rhythm. Bedding was replaced every 3 days, and the experimental animals had free access to food and water in this environment. This experiment was reviewed and approved by the Experimental Animal Ethics Committee of Guizhou Medical University (Approval Number: No2201516).

**Figure 1:**
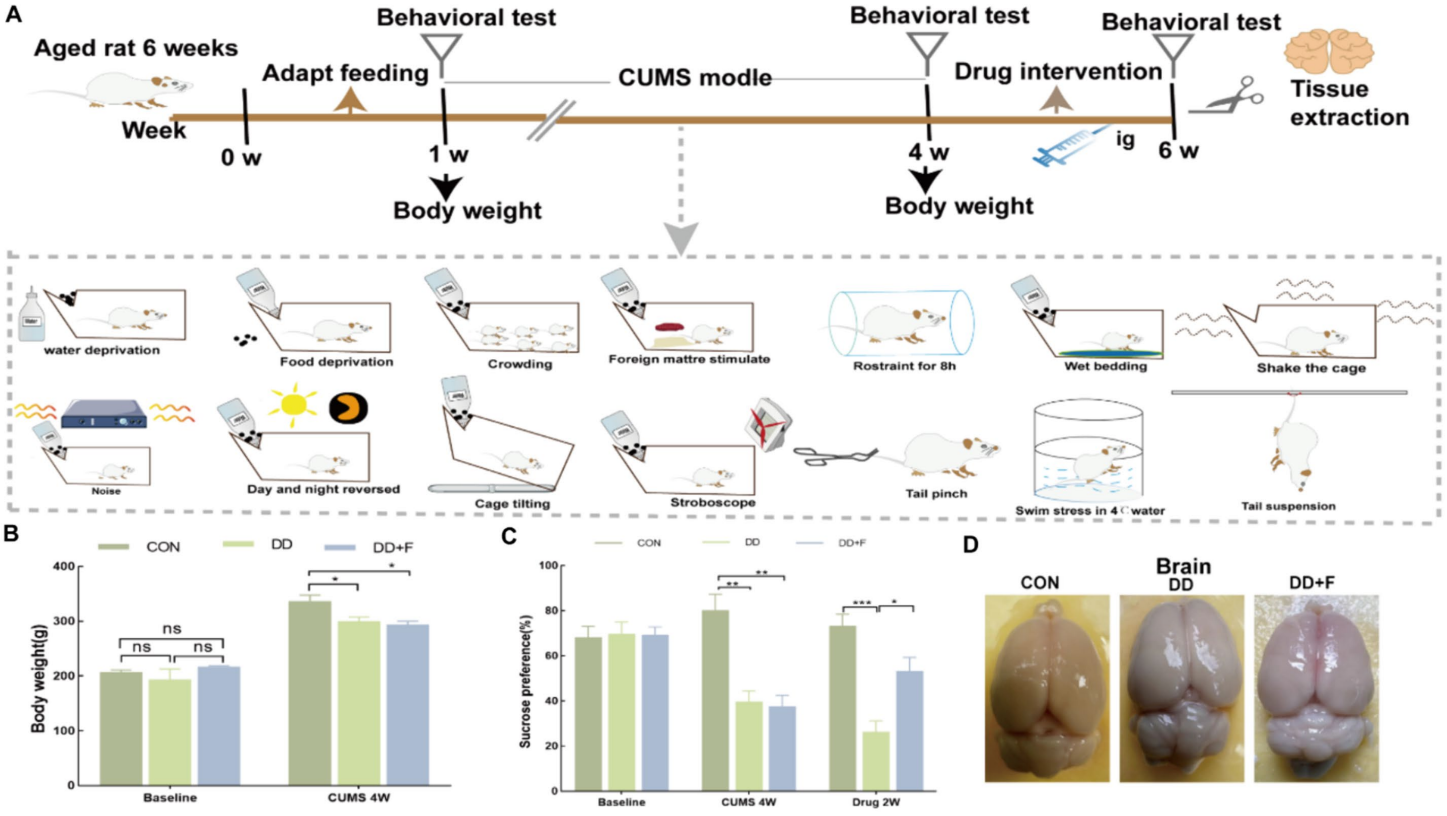
**(A)** Study design of animal experiment. **(B)** Changes in body weight before and after modeling. **(C)** The statistical results of the sucrose preference test. **(D)** Diagram of tissue sampling from each group of rats (*p < 0.05, **p < 0.01, ***p < 0.001, *n* = 5 per group, all data are expressed as mean ± SEM).

Modeling and Grouping: After 7 days of adaptive feeding for 45 rats, the rats were randomly divided into three groups using a random number table: normal control group (CON), depression group (DD), and fluoxetine group (DD + F), with *n* = 15 in each group. The CON group consisted of 5 rats per cage and was maintained under normal feeding conditions. The DD and DD + F groups underwent the chronic unpredictable mild stress (CUMS) combined with social isolation method to induce depression models. In combination with the laboratory conditions, the animals received one type of stimulus randomly each day, with each stimulus being administered 2–3 times on average, ensuring that the animals could not predict the type of daily stimulus. The stressors included: (1) swimming in 4°C ice water for 5 min; (2) damp bedding and dirty cage for 24 h; (3) horizontal shaking cage for 5 min; (4) tail pinching with a sponge clamp 1 cm from the tail root for 1 min; (5) tail suspension for 5 min; (6) water deprivation for 24 h; (7) restraint stress for 8 h; (8) food deprivation for 24 h; (9) noise exposure for 24 h; (10) reversed light–dark cycle (bright light during the night) for 12 h; (11) tilted cage for 24 h; (12) strobe light stimulation for 12 h; (13) unpleasant odor exposure for 24 h; (14) crowding stress for 24 h. After 28 days of modeling and neurobehavioral testing, the DD + F group received fluoxetine 10 mL·kg·d via gastric gavage at 9:00 a.m. every morning for 14 consecutive days, with continuous modeling during the intervention period. The rats in each group were further randomly divided into three batches (5 rats per batch). The first batch was evaluated for neurobehavioral performance before modeling, the second batch after modeling, and the third batch after drug intervention (Figure 1A).

### Macroscopic characteristics

Observe the changes in locomotor activity, neurobehavioral status, hair coat condition, self-grooming behaviors, and stress responses of rats in each group at three time points: pre-modeling, post-modeling, and post-drug intervention.

### Body weight

Record the changes in body weight of rats in each group before and after modeling.

### Behavior tests

#### Sucrose preference test

It is a reward-based test used as an indicator of anhedonia. Anhedonia, or the decreased ability to experience pleasure, is one of the core symptoms of depression (Liu et al., 2018). Steps: (1) Animal Habituation: Experimental rats are placed in the laboratory environment 24 h prior to the test. (2) Drinking Training Period: Lasting for 48 h, during the first 24 h, each rat is provided with two bottles of 2% sucrose solution. In the subsequent 24 h, one bottle remains with 2% sucrose solution, while the other is replaced with pure water (the positions of the two bottles are exchanged halfway through). (3) Testing Phase: After 3 days of adaptive training, the rats are food and water deprived for 24 h before the formal experiment. During the experiment, each rat is simultaneously given a pre-measured amount of 2% sucrose solution and pure water, each in a separate bottle. After 1 h, the positions of the bottles are swapped. Two hours later, the bottles are weighed to calculate the consumption of both the sucrose solution and pure water. The sucrose preference rate of the rats is calculated using a formula:


$$\mathrm{Sucrose Preference Rate}\;\left(\%\right)=\frac{\mathrm{Sucrose Consumption}\;}{\left(\begin{array}{l} \mathrm{Sucrose Consumption}+\\ \mathrm{Pure Water Consumption} \end{array}\right)}\;\times 100\%$$

#### Forced swimming test

The forced swim test is based on the struggling and despairing behaviors exhibited by animals in an inescapable water environment (Crawley, 2008; Ueno et al., 2022). It assesses depressive-like behaviors by observing the immobility of the animals. The steps are as follows: (1) Environmental adaptation and pre-swim sessions are conducted in advance; (2) Formal Experiment: Experimental rats are placed in a circular experimental tank, and software is used to record the total struggle duration, number of struggles, and floating immobility time of the rats for 5 min after immersion.

#### Open field test

It is an experiment designed to study the spontaneous activity and exploratory behavior of animals, which can be used to evaluate their spontaneous activity and anxiety state (Crawley, 2008; Prut and Belzung, 2003; Seibenhener and Wooten, 2015; Walsh and Cummins, 1976). The steps are as follows: (1) Environmental adaptation is conducted beforehand; (2) Formal Experiment: The rat is placed in the open field apparatus with its back facing the experimenter and allowed to freely move for 5 min. The software records the following observation indicators: total horizontal movement distance, time spent in the central zone, number of horizontal crossings, immobility time, time spent in the perimeter zone/total time, perimeter movement distance/total distance, and movement distance in the central area/total distance.

#### Morris water maze

Morris Water Maze (MWM) is a classical behavioral test for assessing spatial learning and memory abilities in rodents (D'Hooge and De Deyn, 2001; Shoji and Miyakawa, 2019). It allows observation of the animal’s learning, spatial orientation, cognitive capabilities, and changes in the process of spatial exploration. The experiment is divided into two phases. The first phase is the place navigation test, lasting for 4 days, with a platform present. The second phase is the spatial probe test, where the target platform is removed on the 5th day, and the total swimming path of the rat within 60 s, the time spent in the target quadrant, the latency to first find the platform (i.e., platform latency), and the number of crossings over the target platform are recorded.

#### Three-chamber sociability test

The aim is to assess the social competence and social novelty deficits of experimental rats (Liu et al., 2015; Lukas and Neumann, 2014; Malhi and Mann, 2018). The procedure is as follows: (1) Adaptation Phase: Rats are placed in a three-chamber apparatus for 10 min prior to the experiment to familiarize themselves with the environment. (2) Social Competence Test Phase: The interaction time between the subject rat and a stranger rat (Stranger 1) as well as an empty restraint device (Empty) is observed. The Social Competence Index is then calculated.


$$\mathrm{Social ability Index}=\frac{\mathrm{Stranger}\;1\;\mathrm{Interaction Time}\;\left(TS1\right)}{\begin{array}{l} \mathrm{Stranger}\;1\;\mathrm{Interaction Time}\;\left(TS1\right)+\\ \mathrm{Empty Cage Interaction Time}\;\left(TE\right) \end{array}}\times 100\%$$

(3) Social Novelty Test Phase: A second stranger rat (Stranger 2) is placed in the empty restraint device. The interaction time between the subject rat and Stranger 1 and Stranger 2 is observed, and the Social Novelty Index is calculated.


$$\mathrm{Social Novelty Index}=\frac{\mathrm{Stranger}\;2\;\mathrm{Interaction Time}\;\left(TS2\right)}{\begin{array}{l} \mathrm{Stranger}\;1\;\mathrm{Interaction Time}\;\left(TS1\right)+\\ \mathrm{Stranger}\;2\;\mathrm{Interaction Time}\;\left(TS2\right) \end{array}}\times 100\%$$

### Hematoxylin-eosin staining

The aim is to observe pathological changes in neurons in the prefrontal cortex of rats. Rats were anesthetized and perfused with 4% paraformaldehyde for fixation and sampling (Figure 1D). The brain tissue was then embedded, sliced, dewaxed, rehydrated, and hydrated. Hematoxylin and eosin (HE) staining kits were used for staining according to the manufacturer’s instructions. Subsequently, rapid dehydration and mounting were performed, and the samples were observed under an optical microscope.

### Nissl staining

The aim is to observe changes in Nissl bodies in neurons of the prefrontal cortex (Lindroos, 1991). The sampling and specimen processing steps are identical to those for hematoxylin and eosin (HE) staining. Subsequently, the specimens are stained with Nissl staining solution (primarily containing toluidine blue), dehydrated, and mounted for observation under a light microscope.

### Hoechst staining

The aim is to observe apoptosis in neurons of the prefrontal cortex. The sampling and specimen processing steps are identical to those for hematoxylin and eosin (HE) staining. A suitable amount of Hoechst 33258 stain (ready-to-use) is then added for staining. After washing, the specimens are observed and photographed under a fluorescence microscope for quantitative analysis.

### Golgi stain

The aim is to observe changes in axonal and dendritic branching of neurons in the prefrontal cortex (De Carlos and Borrell, 2007; Malhi and Mann, 2018; Zhong et al., 2019). Golgi staining was performed using the FD Rapid Golgi Stain Kit, strictly following the manufacturer’s instructions. After staining, conventional gradient ethanol dehydration and xylene clearing were performed, and finally, the specimens were mounted with neutral resin. The prefrontal cortex was observed and photographed under an optical microscope, and ImageJ software was used for quantitative analysis of dendritic spine density.

### Transmission electron microscopy

The aim is to observe the ultrastructure of neurons in the prefrontal cortex. After anesthetizing the rats, perfusion is performed using a specific electron microscopy fixative (2.5% glutaraldehyde buffer). Following sampling, steps including post-fixation, rinsing, dehydration, infiltration embedding, polymerization, slicing, and staining are conducted. Finally, transmission electron microscopy is used to observe the overall, local, and organelle views of the prefrontal cortex slices of the brain tissue, and images are captured and analyzed.

### Western blotting

To measure the relative expression of VGLUT2 protein in the prefrontal cortex of rats. After the final behavioral assessment, the rats were anesthetized, and the prefrontal cortex tissue was collected and stored at −80°C. Protein extraction, BCA protein quantification, gel preparation, electrophoresis, membrane transfer, blocking, primary antibody incubation, secondary antibody incubation, and exposure were performed. Finally, Image J software was used to perform grayscale analysis on the protein bands, and the ratio to the internal reference β-actin was considered as the relative expression of VGLUT2 protein.

### Real-time quantitative polymerase chain reaction

To detect the mRNA expression level of VGLUT2 protein. Sample collection and preservation are the same as those for Western Blot (WB). Total RNA extraction from the prefrontal cortex tissue, RNA concentration and purity measurement, reverse transcription, and RT-PCR reaction are performed. Finally, with β-actin as the internal reference, the relative expression level of mRNA is calculated based on 2^−ΔΔCT^. The primers were designed at NCBI and synthesized by Sheng Gong Biotech (Shanghai) Co., Ltd. The primer sequences are presented in Table 1.

**Table 1:** Real-time quantitative fluorescence PCR primer information.

Gene name		Sequence (5′->3′)	Product length
β-actin	Forward primer	TGTCACCAACTGGGACGATA	165 bp
	Reverse primer	GGGGTGTTGAAGGTCTCAAA	
VGLUT2	Forward primer	CGAGAGACCATCGAGCTGAC	77 bp
	Reverse primer	CCAGAAGAACGACCCGTGAA	

### Enzyme-linked immunosorbent assay

The concentration of VGLUT2 protein was detected using ELISA, with sample collection and storage methods identical to those for Western Blot (WB). The detection was performed strictly according to the instructions provided with the ELISA kit. Finally, the absorbance (OD value) of each well was read at a wavelength of 450 nm.

### Correlation analysis

A correlation analysis was conducted between the histopathological changes indices and the relative protein expression level as well as the mRNA expression level of VGLUT2 in three groups of rats, aiming to investigate whether there is a correlation between neuronal injury and alterations in VGLUT2 protein in experimental rats. Initially, variable data from the three groups of experimental rats were collected. Based on the data type and distribution, Pearson Correlation analysis was employed. Finally, a correlation scatterplot heatmap was generated.

### Statistical analyses

Data processing and analysis were performed using SPSS 26.0 software, and the data were expressed as mean ± standard error. Repeated measures analysis of variance (ANOVA) was used for body weight and neurobehavioral assessments. For the remaining comparisons between groups, if the data followed a normal distribution and had homogeneity of variance, One-way ANOVA was applied, and further pairwise comparisons were conducted using the LSD test. For data that did not meet the criteria for normal distribution or homogeneity of variance, non-parametric tests with rank transformation were used. In correlation analysis, Pearson’s linear correlation analysis was applied for measurement data that conformed to a normal distribution. A *p*-value less than 0.05 was considered statistically significant. Statistical graphs were plotted using GraphPad Prism 9.0, and image collages were created using Adobe Illustrator 2021.

## Results

### Effects on macroscopic characteristics and body weight of experimental rats

Changes in macroscopic manifestations at different time points: During the period of adaptive feeding, rats in all groups exhibited docile temperament, shiny fur, activity, and curiosity, with normal eating and defecation patterns. Following 28 days of stressor stimulation, rats displayed reduced activity, increased sensitivity to provocation, disordered, yellow, and dull fur, as well as softened feces. After 14 days of fluoxetine intervention, conditions improved, with increased activity, more self-grooming behavior, and restoration of fur glossiness.

There was no statistical difference in baseline body weight among the three groups of rats (*p* > 0.05). After 28 days of modeling, compared with the CON group, the body weight gain in the DD and DD + F groups significantly decreased (*p* < 0.05) (Figure 1B).

### Neurobehavioral assessment of rats in each group

Before modeling: There were no statistically significant differences in neurobehavioral indicators in sucrose preference test, forced swimming test, open field test, Morris water maze test, and three-chamber social interaction test (*p* > 0.05).

**Figure 2:**
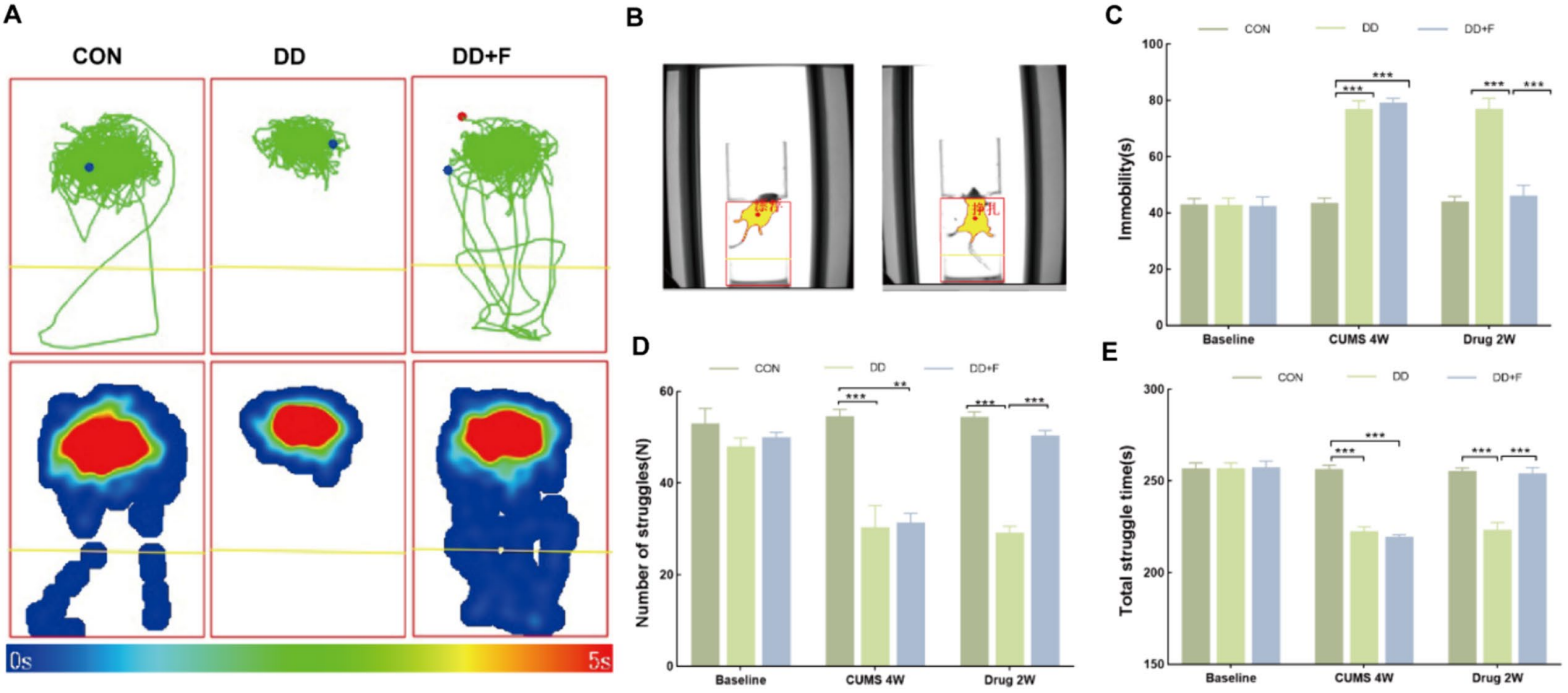
The analysis and statistical results of the forced swimming test. **(A,B)** Forced swim test trajectory map, heatmap, and experimental schematic diagram; **(C–E)** The statistical results of immobility time, struggle times, and total struggle time in the forced swim test (*p < 0.05, **p < 0.01, ***p < 0.001, *n* = 5 per group, all data are expressed as mean ± SEM).

**Figure 3:**
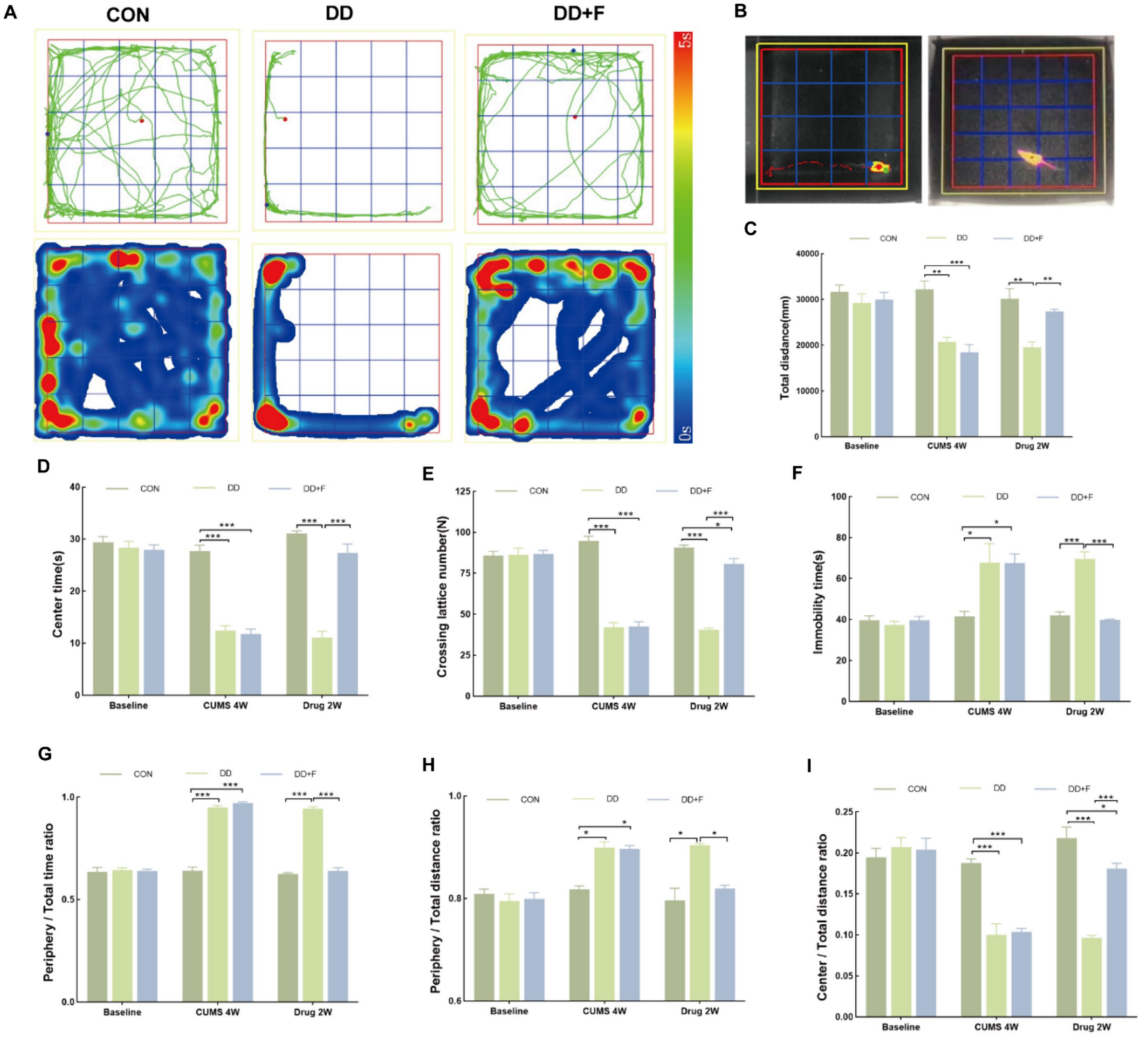
The analysis and statistical results of the open field test. **(A,B)** Trajectory maps, heatmaps, and experimental schematic diagrams of the open field test; **(C–I)** The statistical results of the open field test include the total horizontal movement distance, duration of stay in the central zone, number of horizontal crossings, immobility time, ratio of time spent in the peripheral zone to total time, ratio of peripheral movement distance to total distance, and ratio of movement distance in the central area to total distance (*p < 0.05, **p < 0.01, ***p < 0.001, *n* = 5 per group, all data are expressed as mean ± SEM).

**Figure 4:**
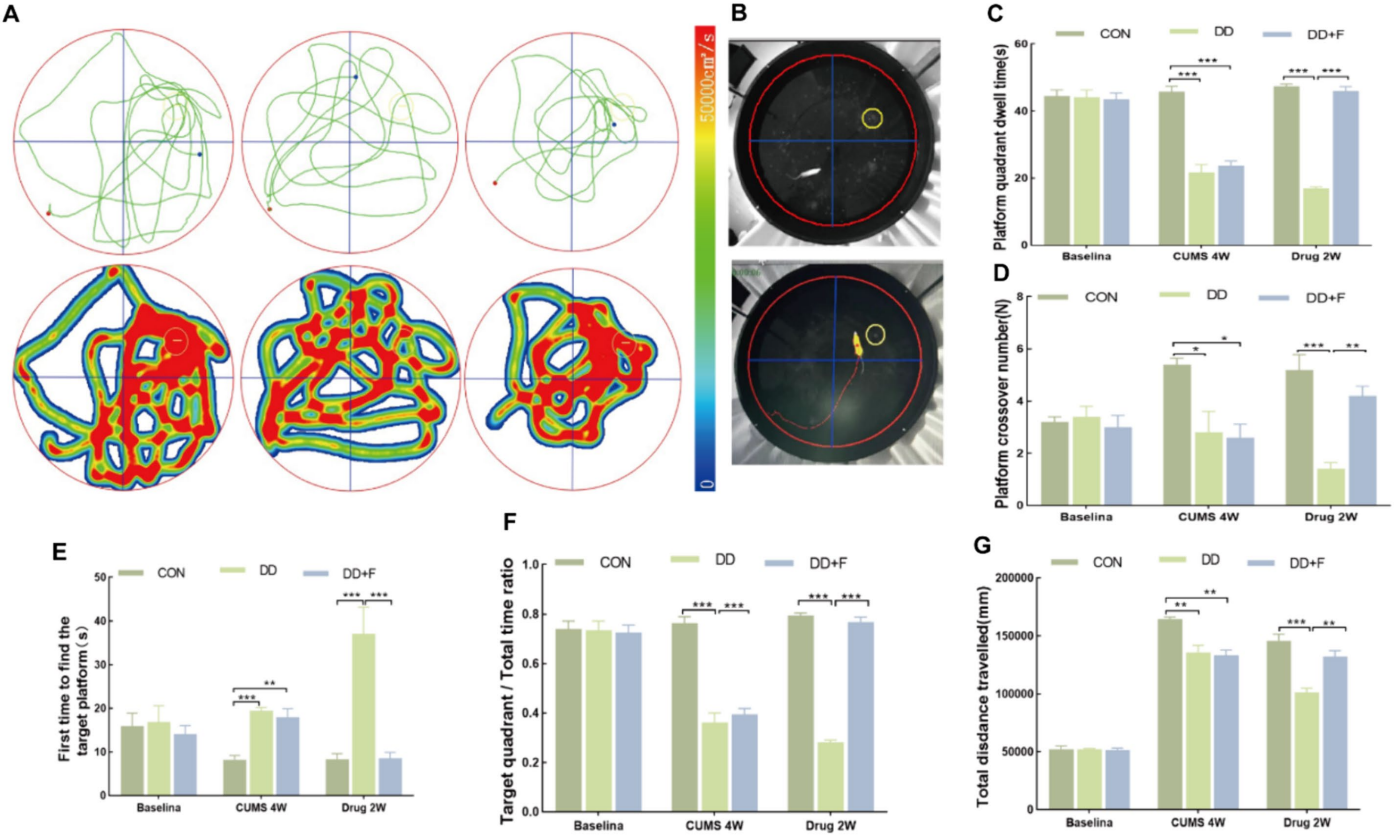
Statistical analysis results of the spatial probe trial phase in the Morris water maze test. **(A,B)** Trajectory maps, heatmaps, and experimental schematic diagrams of the Morris water maze test; **(C–G)** The statistical results of duration of stay in the platform quadrant, number of platform crossings, time to first find the target platform, ratio of duration of stay in the target quadrant to total time, and total distance traveled to explore the platform (*p < 0.05, **p < 0.01, ***p < 0.001, *n* = 5 per group, all data are expressed as mean ± SEM).

**Figure 5:**
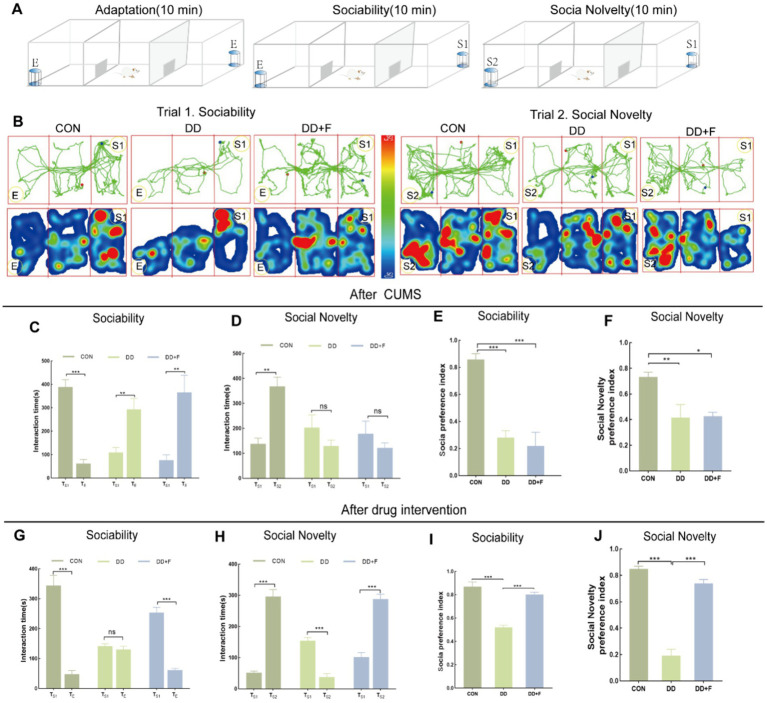
Statistical analysis results of the Three-Chamber social interaction test. **(A,B)** Schematic diagram, trajectory map, and heatmap of the Three-Chamber social interaction test; **(C–F)** The statistical results of the interaction time and social preference index among rats in each group during the social ability testing period after CUMS modeling; and the interaction time and social novelty index among rats in each group during the social novelty testing period. **(G–J)** After fluoxetine intervention, the statistical results of the interaction time and social preference index among rats in each group during the social ability testing period; as well as the interaction time and social novelty index among rats in each group during the social novelty testing period (*p < 0.05, **p < 0.01, ***p < 0.001, *n* = 5 per group, all data are expressed as mean ± SEM).

After modeling: In terms of emotions, compared to the CON group, the DD and DD + F groups showed significantly decreased sucrose preference rates in the sucrose preference test, prolonged immobility time, decreased struggle times, and prolonged total struggle time in the forced swimming test (Figures 2A–E), decreased total horizontal movement distance, shortened retention time in the central zone, reduced horizontal crossings, prolonged immobility time, increased ratio of retention time in the peripheral zone to total time, increased ratio of peripheral movement distance to total distance, and decreased ratio of central zone movement distance to total distance in the open field test (Figures 3A–I). These results indicate that after 28 days of stressor stimulation, rats exhibited behavioral despair, decreased spontaneous motor ability, reduced exploratory behavior, lower curiosity toward new environments, and symptoms of anhedonia. In terms of cognitive and learning abilities: During the spatial exploration phase of the Morris water maze test, compared to the CON group, the DD and DD + F groups showed increased total distance traveled to explore the platform, shortened retention time in the platform quadrant, decreased ratio of target quadrant retention time to total time, increased platform crossings, and prolonged time to first find the target platform (Figures 4A–G). These results indicate that after 28 days of stressor stimulation, rats’ abilities in spatial learning memory acquisition, retention, reproduction, and exploration were impaired. In terms of social abilities: In the second phase of the three-chamber social test for social ability assessment, TS1 was greater than TE in the CON group, while TS1 was less than or equal to TE in the DD and DD + F groups. Compared to the CON group, the DD and DD + F groups showed significantly decreased social ability index and significantly decreased social novelty index (Figures 5A–J). These results indicate that rats’ social abilities were impaired and social novelty was defective after modeling.

After fluoxetine intervention: Compared with the DD group, the neurobehavioral indicators of the DD + F group showed varying degrees of recovery, with an increased sucrose preference rate (Figure 1C). In the forced swimming test, there was a reduction in immobility time, an increase in struggle times, and a prolongation of total struggle time (Figures 2A–E). Additionally, there was an increase in the total horizontal movement distance, an extended retention time in the central zone, an increased number of horizontal crossings, a reduced immobility time, a decreased ratio of retention time in the peripheral zone to total time, a reduced ratio of peripheral movement distance to total distance, and an increased ratio of central zone movement distance to total distance in the open field test (Figures 3A–I). During the spatial exploration phase of the Morris water maze test, the DD + F group exhibited an increased total distance traveled to explore the platform, a prolonged retention time in the platform quadrant, an increased ratio of target quadrant retention time to total time, an increased number of platform crossings, and a shortened time to first find the target platform (Figures 4A–G). Furthermore, the social ability index and social novelty index were significantly elevated in the DD + F group (Figures 5A–J).

In summary, after modeling, depressive model rats exhibited impairments in emotion, cognition, learning, and social abilities. However, after fluoxetine intervention, these symptoms showed varying degrees of recovery.

### Histopathological staining results of the prefrontal cortex in experimental rats

**Figure 6:**
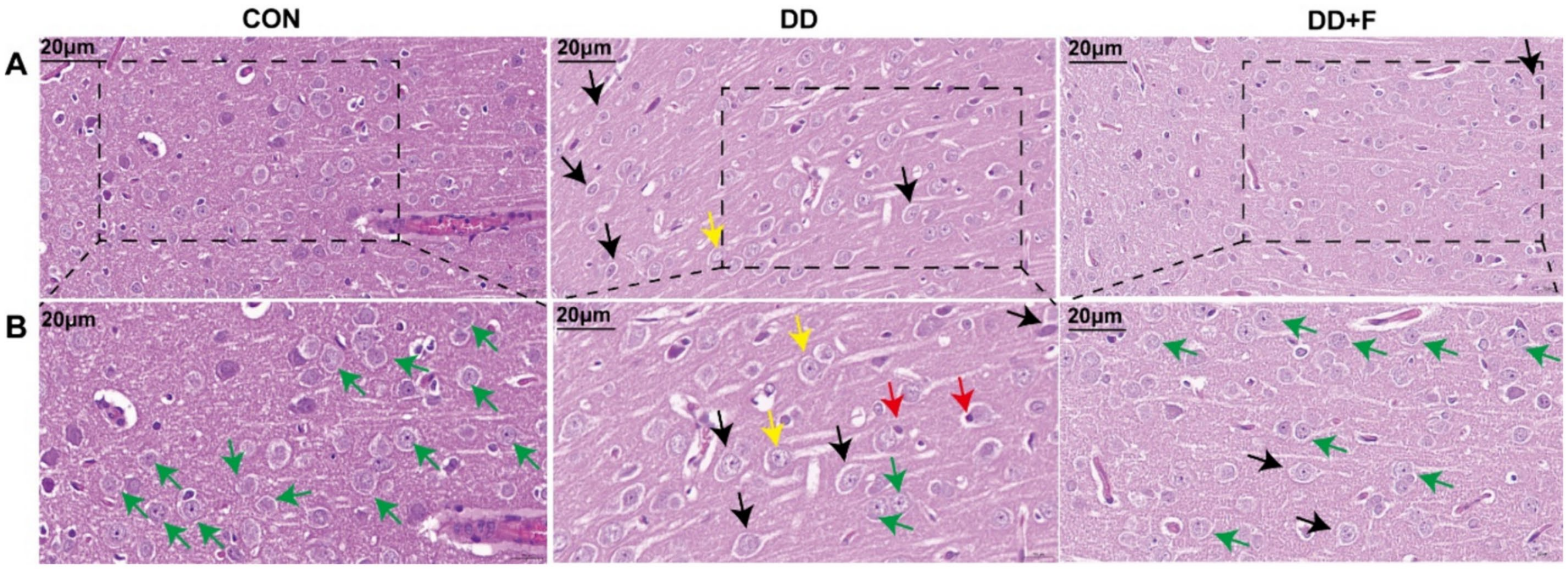
H&E staining results. **(A,B)** are partial views (X400, 20 μm) and enlarged views (X600, 20 μm) of HE staining in rats of each group, respectively. Arrow indications: Green: normal neuronal cells; Black: neuronal cell edema; Yellow: neuronal cytoplasm loose and lightly stained; Red: neuronal nuclear pyknosis and displacement; The rectangular box indicates the locally enlarged area (*n* = 3 per group).

Hematoxylin–eosin staining (H&E): In the CON group, the morphology and distribution of neurons in the prefrontal cortex were normal, with tight alignment, abundant cell density, and clearly centered nucleoli. In the DD group, neuronal damage was most severe, showing disordered cell alignment, sparse distribution, widened gaps, reduced density, decreased number, irregular shape, the appearance of vacuolated cells, and deviated nucleoli. After fluoxetine intervention, neurons in the DD + F group exhibited regular morphology, intact cell membranes, increased cell density, and occasional cell swelling (Figures 6A,B).

**Figure 7:**
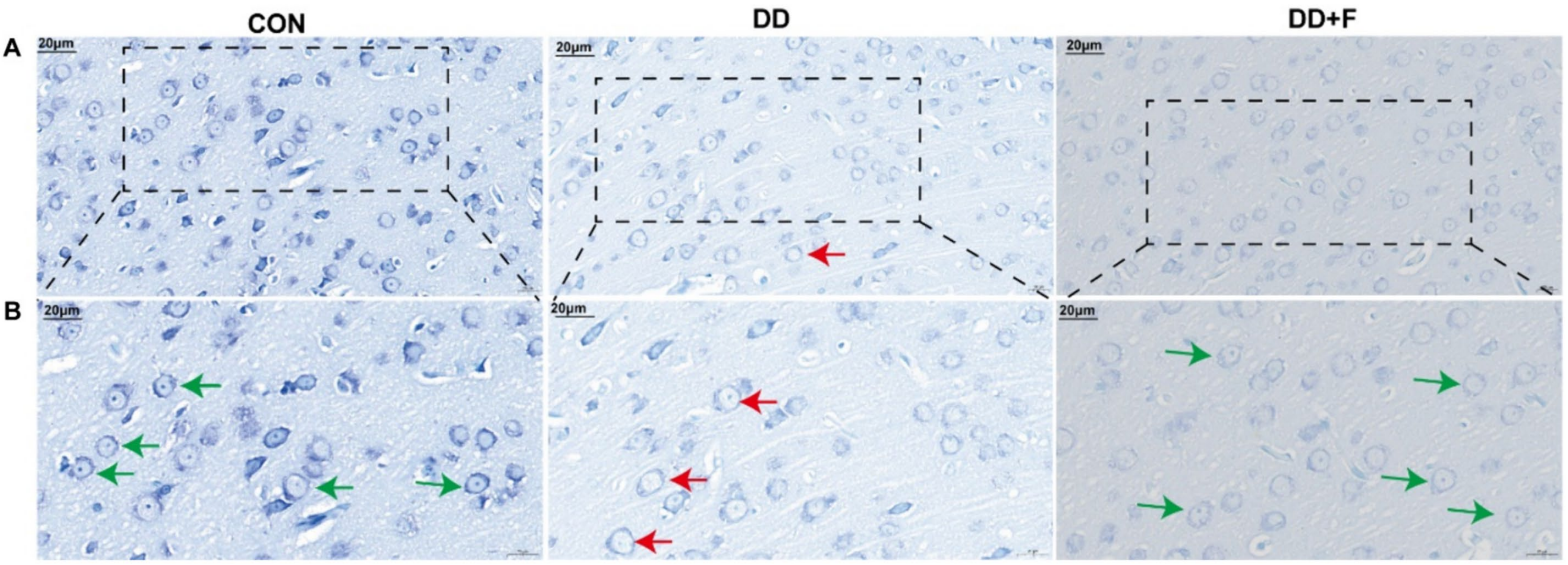
Nissl staining results. **(A,B)** are partial views (X400, 20 μm) and enlarged views (X600, 20 μm) of Nissl Staining in rats of each group. Arrow indications: Green: Neurons with abundant Nissl bodies; Red: Neurons with reduced Nissl bodies. The rectangular box indicates the locally enlarged area (*n* = 3 per group).

Nissl staining: Neurons in the prefrontal cortex of rats in the CON group showed normal distribution, with abundant dark granules or filaments in the cytoplasm, and blue patchy areas in the cytoplasm, representing Nissl bodies. In the DD group, the boundaries of neuronal cells were unclear, and the Nissl bodies around the nucleus were diffusely distributed, with a reduced number or even disappearance. The dissolved part of Nissl bodies appeared as a transparent area. In the DD + F group, Nissl staining in neuronal cells was uniform, with regular morphology, clear contours, distinct boundaries, and increased expression of Nissl bodies (Figures 7A,B).

**Figure 8:**
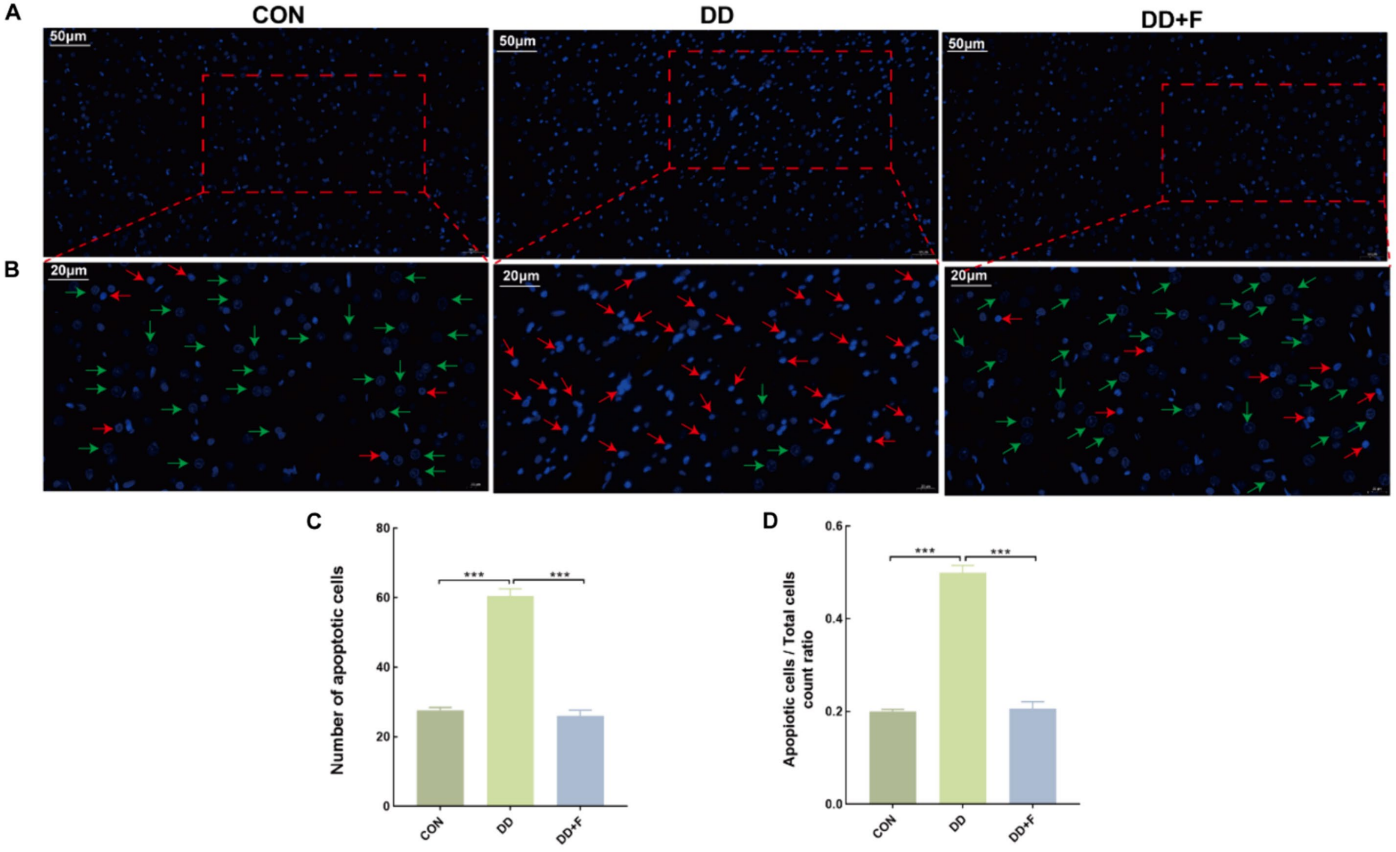
Hoechst staining results. **(A,B)** are partial views (X200, 50 μm) and enlarged views (X400, 20 μm) of Hoechst Staining in rats of each group, respectively. Arrow indications: Green: normal neuronal cells; Red: apoptotic neuronal cells. The rectangular box indicates the locally enlarged area. **(C,D)** Represent the quantitative analysis and statistical chart of neuronal apoptosis through HOECHST staining and the ratio of neuronal apoptosis to total cell count (*p < 0.05, **p < 0.01, ***p < 0.001*, n* = 3 per group, all data are expressed as mean ± SEM).

Effect on Neuronal Apoptosis: Hoechst staining revealed that in the CON group, neuronal cells exhibited uniformly dark blue staining with deeper blue granules inside, and the background was free of any discoloration, with rare areas of pale or bright blue. In the DD group, compared to other groups, the nuclear distribution was sparse, with variable staining intensity. Some nuclei were fragmented, exhibiting enhanced fluorescence in bright blue or pale blue, indicating apoptosis. In the DD + F group, compared to the DD group, the staining was mostly uniform, with increased density and clear boundaries. The staining was primarily light blue, and the occurrence of bright blue and pale blue phenomena was significantly reduced, with occasional apoptotic quadrants. In quantitative analysis, compared to the CON group, the number of apoptotic cells and the ratio of apoptotic cells/total cells in the DD group were significantly reduced (*p* < 0.001). When compared to the DD group, the number of apoptotic cells and the ratio of apoptotic cells/total cells in the DD + F group were significantly increased (*p* < 0.001) (Figures 8A–D).

**Figure 9:**
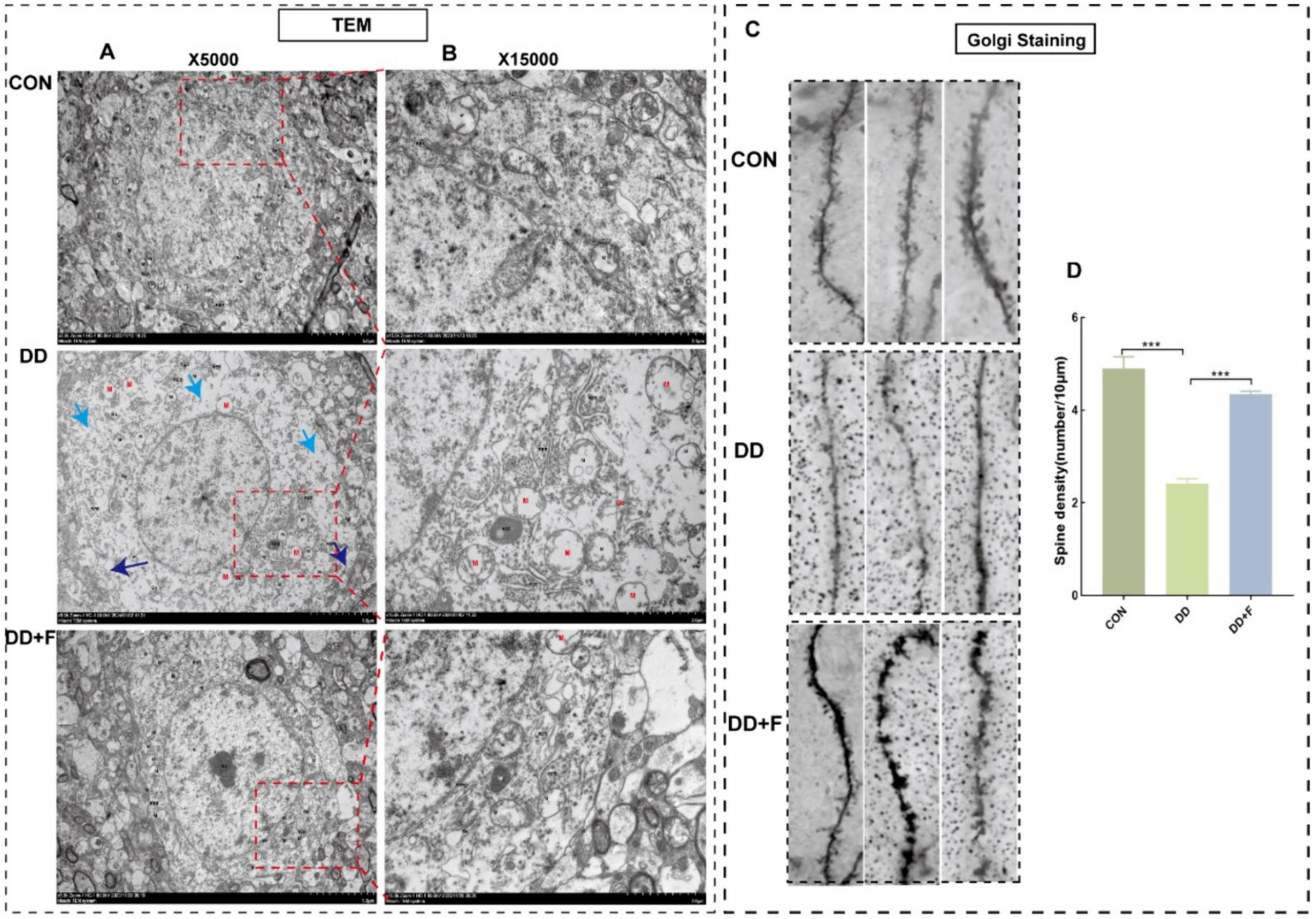
Results of Golgi staining and transmission electron microscopy analysis. **(A,B)** Micrographs of neuronal microstructure in the prefrontal area of rats in each group under transmission electron microscope (X5000) and locally enlarged representative images (X15000). Blue arrow: localized damage to the cell membrane; Cyan arrow: sparse intracellular matrix with decreased electron density; Red: organellar edema. The rectangular box indicates the locally enlarged area. **(C,D)** Representative image of dendritic spine density following Golgi staining (10 μm) and quantitative statistical results. Go, Golgi complex; RER, rough endoplasmic reticulum; M, mitochondrion; ASS, Autophagolysosome; Ly, lysosome (*p < 0.05, **p < 0.01, ***p < 0.001, *n* = 3 per group, all data are expressed as mean ± SEM).

Effects on neuronal dendritic spine density: Golgi staining revealed that compared with the CON group, the dendritic spine density in the DD group was significantly reduced (*p* < 0.05). Compared with the DD group, the dendritic spine density in the DD + F group was increased (*p* < 0.05) (Figures 9C,D).

Alterations in neuronal ultrastructure: Transmission electron microscopy revealed abundant cell bodies, axons, and dendrites in the CON group, with intact overall neuronal cell structure and abundant organelles. In the DD group, neurons in the prefrontal cortex exhibited severe damage, marked edema, incomplete and discontinuous cell membrane structure, local breakage, sparse and dissolved matrix, significantly reduced number of organelles, and the presence of organelle swelling and vacuolization. These injuries were alleviated after fluoxetine intervention (Figures 9A,B).

In summary, after modeling, rats exhibited a decrease in neuronal cells, a reduction in the number and density of Nissl bodies, neuronal apoptosis, damage to neuronal ultrastructure, decreased complexity, and impairment of neuroplasticity. After fluoxetine intervention, these injuries recovered to varying degrees.

### Changes in the concentration, relative expression, and mRNA expression of VGlUT2 protein in rats from different groups

**Figure 10:**
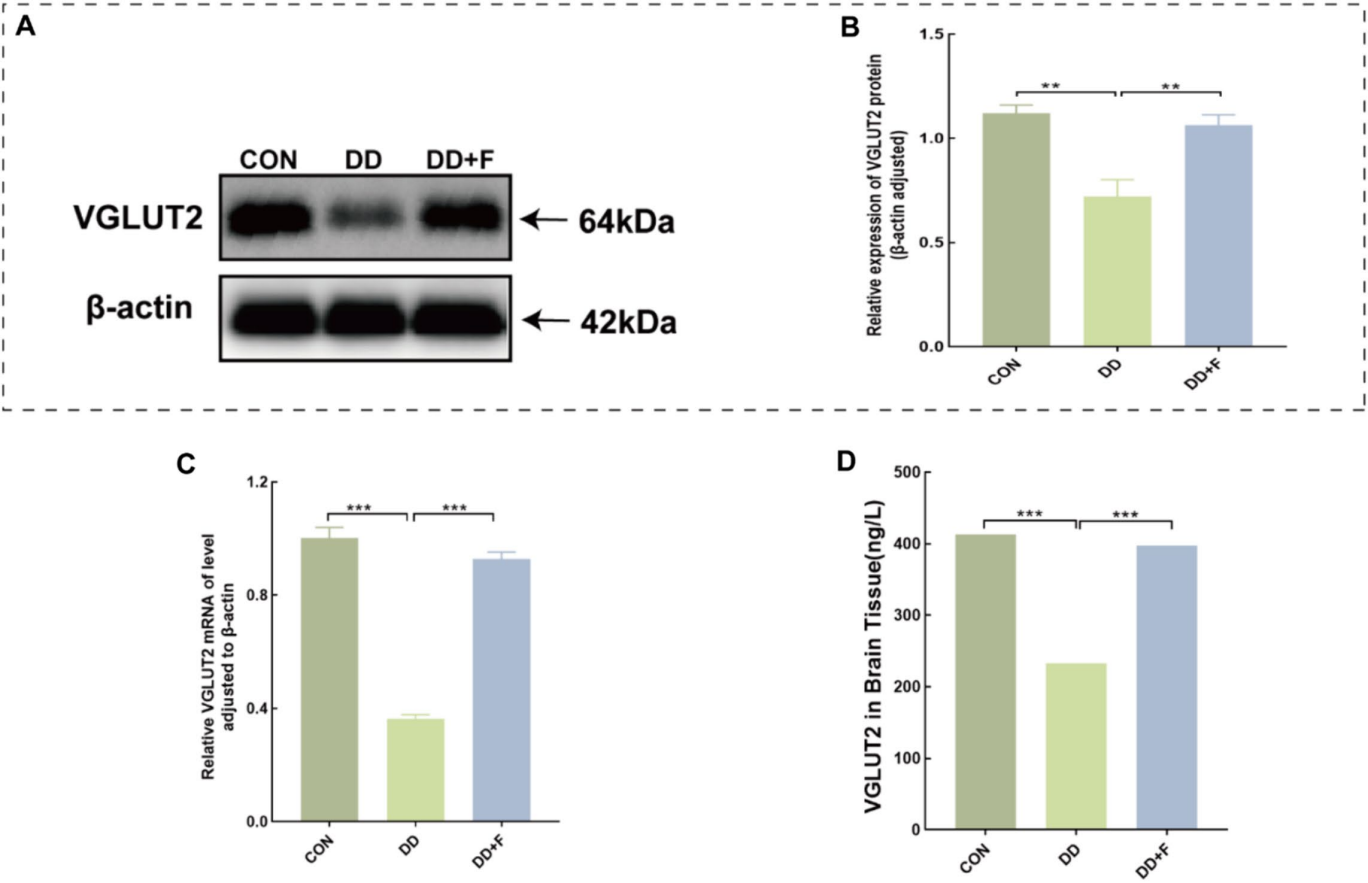
Alterations in VGLUT2 protein in model rats. **(A,B)** Representative results of Western blot analysis showed the levels of VGLUT2 in the brain of rats, Quantification results were normalized against the levels of β-actin. **(C)** Quantitative analysis statistical graph of mRNA expression level of VGLUT2 protein detected by RT-qPCR. **(D)** Quantitative analysis statistical graph of VGLUT2 protein concentration detected by ELISA (*p < 0.05, **p < 0.01, ***p < 0.001, *n* = 3 per group, all data are expressed as mean ± SEM).

WB analysis revealed that the relative expression of VGLUT2 protein in the DD group was significantly lower compared to the CON group (*p* < 0.01). After fluoxetine intervention, the expression level of VGLUT2 protein in the DD + F group was increased compared to the DD group (*p* < 0.01) (Figures 10A,B).

RT-qPCR analysis revealed that the mRNA expression level of VGLUT2 protein in the DD group was reduced compared to the CON group (*p* < 0.001). After fluoxetine intervention, the mRNA expression level of VGLUT2 protein in the DD + F group was significantly increased compared to the DD group (*p* < 0.001) (Figure 10C).

ELISA analysis revealed that the concentration of vGluT2 protein in the DD group was decreased compared to the CON group (*p* < 0.001). After fluoxetine intervention, the concentration of vGluT2 protein in the DD + F group was increased compared to the DD group (*p* < 0.001) (Figure 10D).

### The results of correlation analysis

**Figure 11:**
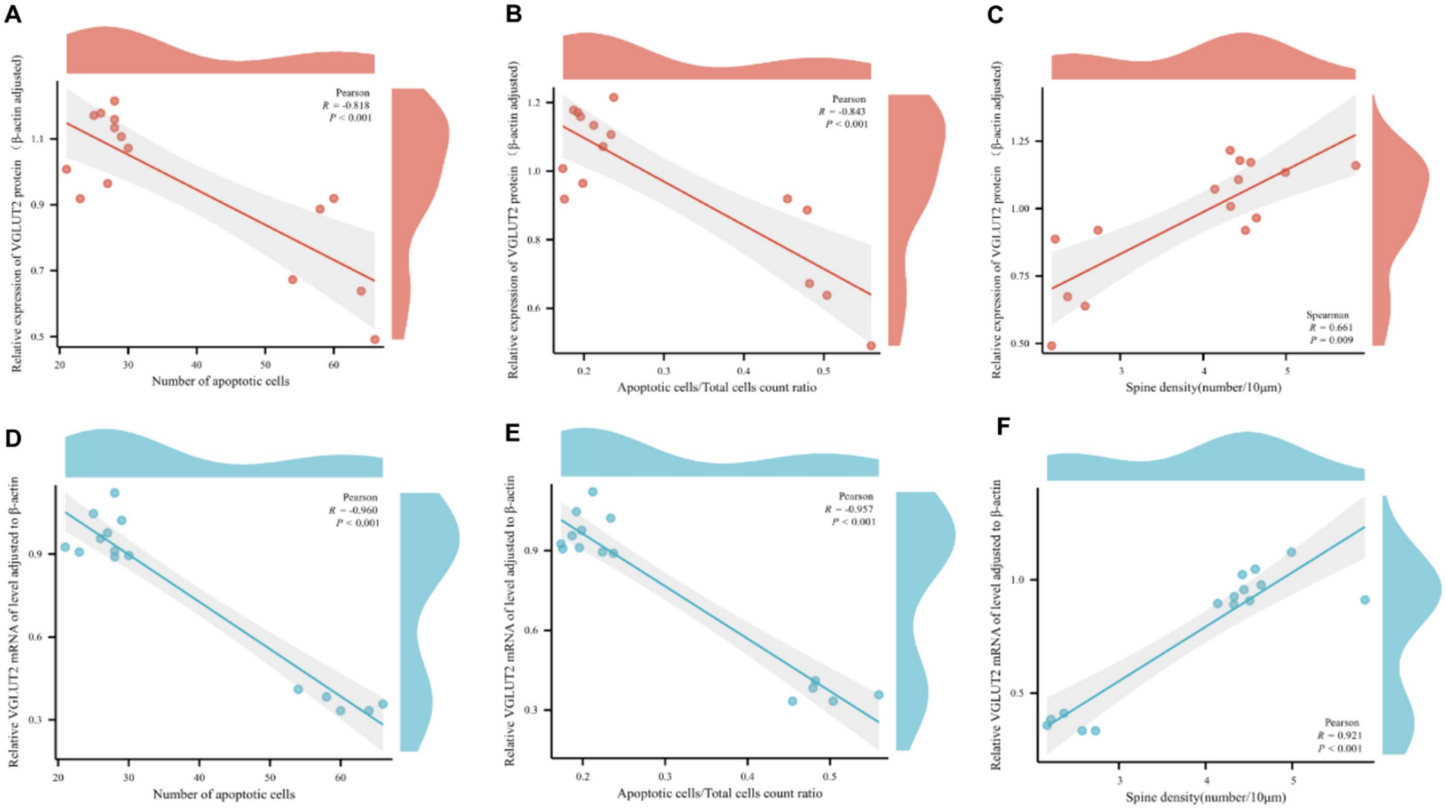
Scatter plot for pairwise variable correlation analysis. **(A–C)** Scatter plot of the statistical results of correlation analysis between the relative expression level of VGLUT2 protein and pairwise variables including the number of apoptotic neurons, the ratio of apoptotic cells to total cells, and dendritic spine density. **(D–F)** Scatter plot of the statistical results of correlation analysis between the mRNA expression level of VGLUT2 protein and pairwise variables including the number of apoptotic neurons, the ratio of apoptotic cells to total cells, and dendritic spine density. In correlation analysis, the correlation coefficient is denoted as *R*. A positive *R* indicates a positive correlation between two variables, while a negative R indicates a negative correlation between two variables. The absolute value of the correlation coefficient represents the degree of correlation, where 0–0.3 represents weak or no correlation; 0.3–0.5 represents a weak correlation; 0.5–0.8 represents a moderate correlation; and 0.8–1 represents a strong correlation.

Correlation analysis was conducted between the relative expression levels of VGLUT2 protein and its mRNA expression levels in rats from various groups and pathological changes. The analysis revealed a significant correlation between the relative expression level of VGLUT2 protein in the prefrontal cortex of rats and pathological changes. Specifically, the expression of VGLUT2 protein showed a significant negative correlation with the number of apoptotic neurons (*R* = −0.818, *p* < 0.001) and the ratio of apoptotic cells to total cells (*R* = −0.843, *p* < 0.001), while it exhibited a positive correlation with dendritic spine density (*R* = 0.661, *p* = 0.009). Similarly, the mRNA expression level of VGLUT2 protein displayed a significant negative correlation with the number of apoptotic neurons (*R* = −0.960, *p* < 0.001) and the ratio of apoptotic cells to total cells (*R* = −0.957, *p* < 0.001), and a positive correlation with dendritic spine density (*R* = 0.921, *p* < 0.001). These findings indicate that in the prefrontal cortex, increased expression of VGLUT2 protein contributes to the repair and improvement of neuronal cell injury and neuroplasticity (Figures 11A–F).

## Discussion

Depression is often accompanied by cognitive dysfunction, especially in terms of selective attention, working memory, and long-term memory (Conradi et al., 2011). The chronic unpredictable mild stress (CUMS) combined with social isolation model, which induces depression through environmental factors and mimics the pathogenesis of depression caused by long-term exposure to multiple stresses and social frustration in humans, has been widely used in the study of depression mechanisms and potential treatments. Through macroscopic characterization, body weight, sugar water preference, and forced swimming test, the behavioral manifestations of depression core symptoms such as anhedonia and despair in model rats are observed. Clinically, depression and anxiety often occur concomitantly. The open-field test is used to observe the rats’ autonomous behavior, anxiety behavior, exploratory behavior, and motor activity. The three-chamber social test is used to observe the social cognitive ability and social novelty of the experimental rats. The Morris water maze test is used to observe the spatial learning and cognitive function of the rats. In the experiment, we performed neurobehavioral tests on the rats after modeling, which was consistent with previous research results (Li et al., 2023).

The prefrontal lobe region undergoes the greatest volumetric changes during evolution, accounting for approximately 30% of the entire cerebral cortex in the human brain. Functionally, the prefrontal lobe is closely related to our emotions, memory, social behavior, and even social status (Zhou et al., 2017). It participates in the physiological and pathological changes caused by stress, serving as a crucial brain region for converting stress into emotional and cognitive disorders, and is considered to be closely related to the occurrence and development of depression (McKlveen et al., 2013). Studies have shown that chronic stress can induce depressive behavior and dendritic morphological changes in prefrontal cortical neurons in rats, and can also lead to cognitive impairment in depressed rats. The neural network of the prefrontal lobe is mainly composed of glutamatergic pyramidal neurons and GABAergic interneurons. Glutamatergic pyramidal neurons account for approximately 80–90% and play a major role in receiving and integrating external information as excitatory neurons in the prefrontal lobe.

Research has indicated that depression patients exhibit a reduction of in glutamate levels in specific brain regions (Fries et al., 2023). Chronic stress and depression can lower the glutamate and GABA neurotransmitter systems in cortical and limbic brain regions. Ketamine can rapidly upregulate the GABA and glutamate neurotransmitter systems, reversing the effects of chronic stress exposure (Duman et al., 2019). Glutamate, the excitatory neurotransmitter, is also significantly associated with cognitive impairment, and increased biosynthesis of glutamate can improve cognitive decline (Li et al., 2024). Vesicular glutamate transporter 2 (VGLUT 2), a classic marker of excitatory neurons, also known as DNPI and SLC17A6, specifically loads glutamate into synaptic vesicles and promotes its release, serving as a classic marker of glutamatergic nerve terminals and glutamatergic synapses (Chmielewska et al., 2019). Studies have shown that VGLUT2 neural activity can regulate depression-like behaviors (Luo et al., 2023), suggesting that the expression of VGLUT2 is involved in the changes in emotional, behavioral, and cognitive functions in depression.

The results of this study indicate that after 4 weeks of CUMS combined with solitary housing modeling, depression model rats exhibited depressive-like behaviors, as well as impairments in social, cognitive, and learning abilities, which were consistent with the criteria for a cognitive dysfunction model of depression and aligned with relevant research findings (Li et al., 2023). Concurrently, pathological damage was observed in the prefrontal cortex neurons of depression model rats, accompanied by a decrease in neuronal plasticity and complexity. The expression of VGLUT2 protein and its mRNA was downregulated, and the concentration of VGLUT2 protein decreased. Following intervention with SSRIs medication, fluoxetine, improvements in emotional, social, cognitive, and learning abilities were observed in the depression model rats using neurobehavioral testing. Histopathological analysis revealed varying degrees of recovery from pathological damage in the prefrontal cortex. Transmission electron microscopy and Golgi staining revealed improvements in neuronal ultrastructure and neuroplasticity damage. Western blot (WB), reverse transcription-quantitative polymerase chain reaction (RT-qPCR), and enzyme-linked immunosorbent assay (ELISA) indicated increased relative expression levels of VGLUT2 protein, mRNA expression levels, and protein concentration. Correlation analysis found a negative correlation between pathological damage in the prefrontal cortex and the expression of VGLUT2 protein and its mRNA, and a positive correlation with dendritic spine density, suggesting that the expression of VGLUT2 protein significantly affects pathological repair and plasticity of prefrontal cortex neurons. The antidepressant and cognitive-improving effects exerted by VGLUT2 upregulation may provide a new research perspective for cognitive dysfunction in depression.

The results of this study suggest that CUMS combined with isolation rearing may lead to increased neuronal apoptosis, ultrastructural damage, and decreased neural plasticity and complexity in the prefrontal cortex of rats. VGLUT2 may participate in the development of depression with cognitive dysfunction by regulating pathological changes in the prefrontal cortex. VGLUT2 might improve cognitive function in depressed rats by exerting neuroprotective effects.

## Data Availability

The raw data supporting the conclusions of this article will be made available by the authors, without undue reservation.
